# Tachykinin receptors antagonism for asthma: a systematic review

**DOI:** 10.1186/1471-2466-11-41

**Published:** 2011-08-02

**Authors:** Renata Ramalho, Raquel Soares, Nuno Couto, André Moreira

**Affiliations:** 1 Department of Immunology, Faculty of Medicine, University of Porto, Porto, Portugal; 2 Department of Biochemistry, Faculty of Medicine, University of Porto, Porto, Portugal; 3 Department of Immunoallergology, Hospital of São João E.P.E., Porto, Portugal; 4 Portuguese Oncology Institute IPOPFG-E.P.E, Porto, Portugal; 5 Centro de Dermatologia Epidermis, Instituto CUF, Porto, Portugal

## Abstract

**Background:**

Tachykinins substance P, neurokinin A and neurokinin B seem to account for asthma pathophysiology by mediating neurogenic inflammation and several aspects of lung mechanics. These neuropeptides act mainly by their receptors NK1, NK2 and NK3, respectively which may be targets for new asthma therapy.

**Methods:**

This review systematically examines randomized controlled trials evaluating the effect of tachykinins receptors antagonism on asthma. Symptoms, airway inflammation, lung function and airway inflammation were considered as outcomes. We searched the Cochrane Airways Group Specialized Register of Asthma Trials, Cochrane Database of Systematic Reviews, MEDLINE/PubMed and EMBASE. The search is as current as June 2010. Quality rating of included studies followed the Cochrane Collaboration and GRADE Profiler approaches. However, data were not pooled together due to different measures among the studies.

**Results:**

Our systematic review showed the potential of NK receptor antagonist to decrease airway responsiveness and to improve lung function. However, effects on airway inflammation and asthma symptoms were poorly or not described.

**Conclusion:**

The limited available evidence suggests that tachykinin receptors antagonists may decrease airway responsiveness and improve lung function in patients with asthma. Further large randomized trials are still required.

## Background

A sharp increase in the prevalence, morbidity, mortality, and economic burden associated with asthma over the last 40 years, particularly in children is occurring. Approximately 300 million people worldwide have asthma, and its prevalence increases by 50% every decade [1]. Because no asthma definition exists, an operational definition was proposed by the Global Initiative for Asthma: a chronic inflammatory disorder of the airways associated with airway hyperesponsiveness that leads to recurrent episodes of wheezing, breathlessness, chest tightness, and coughing [1]. Therefore, asthma is a phenotypically heterogeneous disorder and, over the years, many different clinical subtypes of asthma have been described.

Recently, a model of interaction between different pathophysiologic mechanisms known to affect asthma phenotype was suggested [2]. This is of particular importance not only to recognize asthma as a complex disease for which different endogenous and exogenous factors may account, but also for emphasising the need of a precise definition of the asthma phenotype as a tool for improved asthma care. Despite major advances in understanding the pathogenesis of asthma and improvements in asthma drugs, the accompanying benefits have been less than expected. Drug strategies for asthma have been based on the premise that symptoms derive directly and immediately from airway inflammation focusing on the development of anti-inflammatory drugs, particularly steroids that show broad-spectrum inhibitory activity against a wide range of effector cells and their products. Evidence for an interaction between chronic inflammation and neural dysfunction points to an involvement linking the nervous and the immune system in the airways [3]. In this context, neuropeptides and neurotrophins have been recognized as key mediators of neuro-immune interactions [3] and investigation regarding the development of pharmacological compounds specifically targeting these molecules could be of interest in asthma.

Tachykinins include a family of neuropeptides with a wide range of actions in human body [4]. The most relevant are substance P, neurokinin A (NKA) and neurokinin B (NKB) and act mainly by their receptors which are NK1, NK2 and NK3, respectively [5]. Interestingly, tachykinins are potent mediators of a number of functions in the airways [6]. Within human airways, substance P and NKA are the predominant neuropeptides released from nonadrenergic-noncholinergic system by mechanical, thermal, chemical or inflammatory stimuli. NK3 receptors have been only recently considered in studies of airway regulation in health and in disease [7]. This is due to the observation that NKB, the most potent endogenous ligand for the NK3 receptor, is not readily localized to the airway nerves. In addition, contrasting the effects of NK1 and NK2 receptor activation in the airways, which induce pronounced and thus readily quantifiable effects in the lungs (e.g. bronchospasm, vasodilatation, vascular leakage, mucus secretion), the actions of NK3 receptor-selective agonists are mostly subtle and not simply measured with commonly used airway function measures [7].

Recent findings point to tachykinergic systems as promising targets of novel clinical agents. In asthma, the modulation of their receptors appear to influence a variety of pathological symptoms and processes such as inflammation [4]. However, improved therapeutic strategies can only be delineated if clinical effects are achieved by well-designed randomized controlled trials. Therefore, in an effort to assemble the clinical effects of tachykinin receptors modulation on asthmatic patients the authors conducted this systematic review.

## Methods

### Studies, participants, interventions and outcomes

The review was restricted to randomized controlled clinical trials and controlled trials, which studied the effect of tachykinin receptor antagonists on asthma related outcomes. Participants were adults or children with a medical diagnosis of asthma. All health-care settings were considered eligible and no restrictions for gender were made. The selected outcome measures were: symptoms, adverse events of the treatments, lung function, airway responsiveness and inflammation.

### Search strategy

An initial search was carried out using the Cochrane Airways Group Specialized Register of asthma trials and EBM Reviews - Cochrane Database of Systematic Reviews. Additional and separate searches were carried out on MEDLINE (1966-2010), EMBASE (1980-2010) and PubMed. The subject-specific search terms were "tac1r", "tac2r" "tac3r", "tachykinins", "tachykinins antagonists" "NK-1R", "NK-2R", "NK-3R", "substance P", "neurokinin A", "neurokinin B", "asthma". The reference lists of all primary studies were reviewed to identify trials not captured by electronic and manual searches. All titles and abstracts identified by the search that appeared relevant were selected for full text review. Update searches have been run up to June 2010.

### Data collection and analysis

Abstracts of articles identified were viewed and articles that appear to fulfil the inclusion criteria were retrieved in full. Two reviewers (RR and NC) applied the inclusion criteria independently and where disagreement occurred they were resolved by consensus. Data were extracted by one reviewer (RR) and checked by other (AM). The methodological quality of the included trials was assessed with particular emphasis on allocation concealment and randomization, which was ranked using the Cochrane Collaboration and GRADE Profiler approaches [8]. This systematic review was in agreement with the PRISMA guidelines [9]. Meta-analysis was not conducted due to small number of included trials and heterogeneity across studies.

## Results

**Figure 1:**
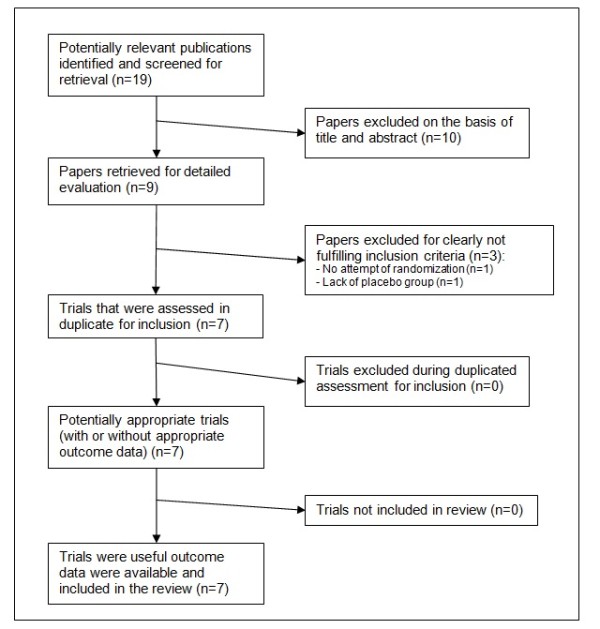
**Flowchart of selection process**.

Based on title, nineteen studies were identified as potentially relevant. However, based on abstract, only seven studies appeared to meet the inclusion criteria and their full text was obtained (Figure 1). Therefore, we included 7 studies comprising the use of five tachykinin receptors antagonists: NK-2 in two [10-12], NK-1/NK-2 in four [11,13-15] and NK-1/NK-2/NK-3 in other one [16], referring to a total of 98 subjects (Table 1). All studies used different doses of tachykinin receptors antagonists. Regarding treatment dosages, 5 studies used single-doses [11-13,15,16] while in 2 studies doses of antagonists were given during 2 and 9 consecutive days [10,14]. Antagonists were administered by inhalation in 3 studies [10,15,16] and orally in 4 studies [11-14]. None of the studies reported changes in asthma symptoms as an outcome.

**Table 1:** Included studies on the effect of tachykinin receptors antagonism on asthma

Study/reference	Design and participants	Intervention	Outcomes	Results
Shelfhout *et al*., 2009 [11]	• Single-center, RCT, double-blind, crossover • 12 non-smoking subjects males	• 2 or 8 mg of MEN 48968 (dual NK-1/NK-2 receptor antagonist) single inhaled dose	• Airway responsiveness to NK antagonist	• Decreased airway responsiveness
Boot et al., 2007 [13]	• Single-center, RCT, double-blind, crossover • 19 non-smoking subjects (15 females);mean age 30 yr	• 4.8 mg of AVE 5883 (dual NK-1/NK-2 receptor antagonist) single inhaled dose	• Airway responsiveness to NK antagonist • Lung function • Airway inflammation	• Decreased airway responsiveness • Inhibition of bronchoconstriction
Schelfhout *et al*., 2006 [16]	• Multi- center, RCT, double-blind, crossover • 15 subjects (7 females); mean age 30 yr	• 200 mg CS-003 (triple NK-1/NK-2/NK-3 receptor antagonist) single oral dose.	• Airway responsiveness to NK antagonist • Lung function	• Decreased airway responsiveness
Joos *et al*., 2004 [15]	• Multi- center, RCT, double-blind, crossover • 18 males; mean age 28 yr	• 100 mg DNK 333 (dual NK-1/NK-2 receptor antagonist) single orally dose.	• Airway responsiveness to NK antagonist • Lung function	• Decreased airway responsiveness
Kraan *et al*., 2001 [10]	• Single-center, RCT, double-blind, crossover • 12 atopic non-smoking males; mean age 30 yr	• 100 mg (4 × 25 mg) SR 48968C (NK-2 receptor antagonist) orally once-daily during 9 days.	• Airway responsiveness to NK antagonist • Lung function	• No effect
Schoor *et al*.,1998 [12]	• Single-center, RCT, double-blind, crossover • 12 non-smoking males; mean age 29 yr.	• 100 mg SR 48968 (NK-2 receptor antagonist) orally single dose	• Airway responsiveness to NK antagonist	• Decreased airway responsiveness
Joos *et al*., 1996 [14]	• Single-center, RCT, double-blind, crossover • 10 atopic non-smoking subjects (2 females); mean age 27 yr	• 4 mg FK224 (dual NK-1/NK-2 receptor antagonist) inhaled once-daily for 2 days.	• Airway responsiveness to NK antagonist • Lung function	• No effect

**Summary**	• 98 young adults (24 females, 74 males)	• Seven different antagonists acting on NK-1R, NK-2, NK-3 in mono, dual or triple combinations.	• Airway responsiveness to NK antagonist, lung function and airway inflammation.	• Five studies showed positive effects on airway responsiveness and/or lung function and two studies failed to demonstrate effects on asthma outcomes.

**Figure 2:**
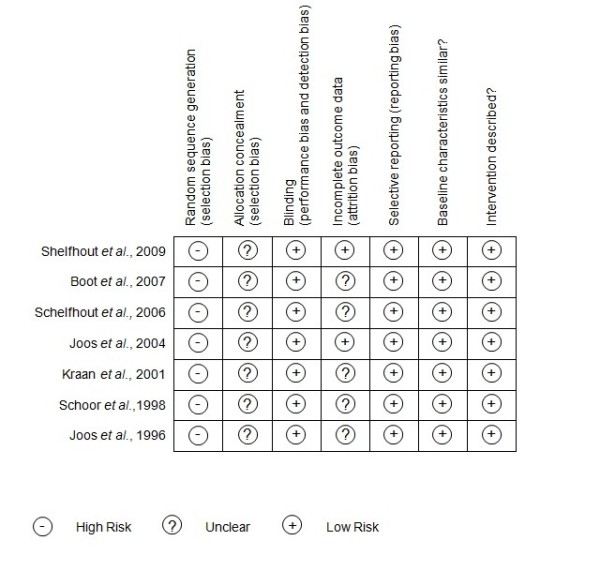
**"Risk bias" summary: reviewer author's judgements about each risk of bias item for included studies**.

**Figure 3:**
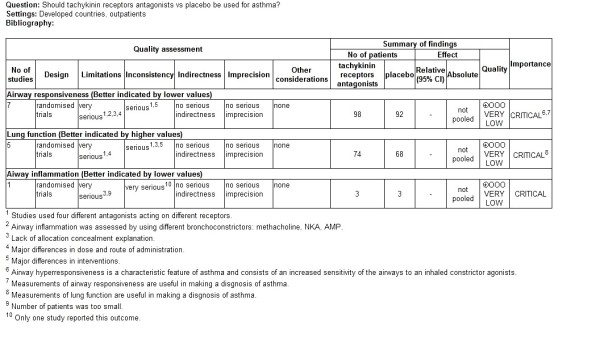
**Tachykinin receptors antagonists compared to placebo for asthma grading evidence**.

Reviewer's judgments about risk of bias for each study was summarized in Figure 2. As shown in Figure 3, methodological quality of the included trials was graded as very low (Figure 2). Data extraction from included studies was summarized in Table 2.

**Table 2:** Effect of tachykinin receptors antagonism on asthma outcomes

		Control		Treated	
**Outcome**	**N**	**Mean(SD)**	**N**	**Mean(SD)**	***p***

• **AIRWAY RESPONSIVENESS:**					
**Log PC_35 _sGaw NKA mol/ml**					
FK224 (NK-1R/NK-2R antagonist)	10	-6.04(0.18)	10	-6.19(0.23)	*n.s*.
Joos et al, 1996					
**PC_20_-AMP mg/ml**					
SR 48968C (NK-1R/NK2R antagonist)					
Kraan et al, 2001	12	1.46(1.11)	6	1.25(0.98)	*n.s*.
**Log_10_PC_20_-NKA mol/ml**					
MEN 11420 (NK-2R antagonist)					
Shelfhout et al, 2009	12	-6.38(0.26)	12	-6.92(0.25)	0.028
AVE 5883 (NK-1R/NK-2R antagonist)					
Boot et al, 2007	19	-6.40(0.12)	19	-6.74(0.12)	0.004
CS-003 (NK-1R/NK-2R/NK-3R antagonist)					
Schelfhout et al, 2006	15	-5.92(0.83)	15	-6.99(0.66)	<0.05
DNK 333 (NK-1R/NK-2R antagonist)					
Joos et al, 2004	18	-5.60*	18	-6.8*	<0.001
SR 48068 (NK-2R antagonist)					
Schoor et al, 1998	12	-7.02(0.28)	12	-7.64(0.19)	0.05

• **LUNG FUNCTION:**					
**FEV_1 _(% fall)**					
AVE 5883 (NK-1R/NK-2R antagonist)					
Boot e tal, 2007	19	145.5(11.7)	19	116.2(11.7)	0.01
SR 48968C (NK-1R/NK2R antagonist)					
Kraan e tal, 2001	12	81(4)	6	78(3)	*n.s*.
**FEV_1 _(L)**					
CS-003 (NK-1R/NK-2R/NK-3R antagonist)					
Schelfhout e tal, 2006	15	3.34(0.62)	15	3.25(0.71)	*n.s*.
FK224 (NK-1R/NK-2R antagonist)					
Joos e tal, 1996	10	3.66 × 10^-3^(0.25)	10	3.67 × 10^-3 ^(0.21)	*n.s*.

• **AIRWAY INFLAMMATION:**					
**Eosinophils %**					
AVE 5883 (NK-1R/NK-2R antagonist)					
Boot e tal, 2007	19	4.86(1.75)	19	3.33(1.58)	*n.s*.
**eNO ppb**					
AVE 5883 (NK-1R/NK-2R antagonist)					
Boot e tal, 2007	19	37.64(6.40)	19	43.44(6.57)	0.32

* 95% Confidence Interval for the difference in Log_10_PC_20_-NK, 0.841-1.616.

SP - substance P; eNO - exhaled nitric oxide; *n.s*. - reported as not significant.

### Airway responsiveness

Five of the seven studies reported a decreased in airway responsiveness with the use of tachykinin receptors antagonists. However, this effect seemed to be dose dependent since in the study from Shelfhout the improvement of this outcome was not seen for the lowest dose tested [11]. These studies show, first, these drugs are indeed tachykinin receptors antagonists, and secondly, given the very modest shifts in the concentration response curves, nearly all of these drugs were underdosed.

### Lung Function

Four studies reported lung function as an outcome. All but one failed to show significant effects with tachykinin receptors antagonists treatments [10,14-16]. However, in the study from Boot *et al. *[13] NK-1/NK-2 antagonist administration reverted the NKA-induced bronchoconstriction.

### Airway inflammation

Only one study reported airway inflammation as an outcome [13]. In this study, authors concluded that airway inflammation evaluated either by changes in exhaled nitric oxide or sputum cells counts was not improved after a dual NK1/NK2 antagonist [13].

### Adverse events

Adverse events were only considered as an outcome in three studies [11,13,15]. In the study from Boot *et al. *[13] adverse events were reported as transient self-limiting bronchospasm starting within 12 minutes after drug inhalation (8 patients under NK-1/NK-2 antagonist and 4 patients under placebo), headache (5 patients under NK-1/NK-2 antagonist and 3 patients under placebo), and self-limiting dyspneia (2 patients under NK-1/NK-2 antagonist and 5 patients under placebo). Shelfhout *et al. *[11] reported no serious adverse effect with the NK-1/NK-2 antagonist studied. In the study from Joos *et al. *[15] was reported that 4 patients referred fatigue, headache, aggravated asthma, cough and wheezing. However, as no changes in laboratory parameters were observed authors concluded that these events were not related to the NK-1/NK-2 antagonist tested.

## Discussion

Our systematic review showed: first, the use of NK receptor antagonist decreases airway responsiveness and may improve lung function; secondly effects on airway inflammation and asthma symptoms have been poorly or not addressed at all. It must be noted that these conclusions are limited by different interventions, doses, route of administration, schedules and samplings, as well as patient characteristics. Nevertheless, our review was strengthened by the use of a complete search strategy and the omission of relevant studies seems unlikely.

The most important limitation of our review was the relatively small number of well-designed, adequately powered studies reporting all outcomes of interest. Additional limitations were: each of seven studies used a different drug, most of these studies failed to document adequate dosing, and nearly every study targeted a different combination of the NK1, NK2 and NK3. The effectiveness of tachykinin receptors antagonism on cough and chest tightness was not reported. Moreover, airway inflammation was only discussed in one study. As an inflammatory condition, evaluation of the effect of drugs in airway inflammation is a major outcome in asthma. Few studies analysed drug safety and reported side effects. However, the undesired effects observed seemed to be not related to the use of antagonists. Synthesis of the literature with such few studies was difficult because a single study may affect the outcomes. In this situation, heterogeneity may be introduced and publication bias may be not easy to detect.

Selective tachykinin receptors antagonists tested on guinea pigs have been shown to inhibit the late allergic and airway hyperesponsiveness and reduce eosinophilic infiltration and vascular permeability [8,17,18]. These models showed promising effects of dual and triple antagonists in reducing cough and vascular hyperpermeability and inhibiting airway hyperesponsiveness and bronchoconstriction. Similar results were observed in studies using ferrets and mice providing additional evidence in reducing mucus production [3,19]. However, as for other studies involving animals, the extrapolation for humans has been difficult.

Playing substance P the more relevant role in allergen-induced airway inflammation, the lack of effectiveness in reducing airway hyperesponsiveness may be due to compromising of results by testing antagonists' protection against NKA inhalation. It is recognized that substance P, acting through its NK-1 receptor, induces bronchoconstriction and airway hyperesponsiveness, with asthmatics being more sensitive [5,15]. Acting through its NK-2 receptor, inhaled NKA had been shown to be more responsible for cause bronchoconstriction [16]. Lack of efficacy in other studies may also be explained by requirement of different concentrations of antagonists to inhibit bronchoconstriction.

The use of tachykinin receptors antagonists in dual or triple combination rather than one antagonist alone may be regard as a more efficient way of improve asthma outcomes. However, the use of these antagonists in combination does not allow us to determine the relative contribution of each tachykinin receptor subtype to bronchoconstrictor effect of NKA and SP in asthma. In fact, these receptors have very different effects on airway cells (Table 3), airway vascular smooth muscle and central nervous system reflexes. Future investigation should compare the use of NK-1 and NK-2 receptors antagonists alone and in combination in order to set the relative contribution of NKA and SP antagonism in asthma. Furthermore, it seems imperative to gain more insight into the role of tachykinin receptors pathways as well as into the effectiveness of their antagonisms on asthma related symptoms like cough and chest tightness.

**Table 3:** Effects of tachykinins receptors NK1, NK2 AND NK3 on human airway cells

		Receptors	
**Effect on human airway cells**	**NK1**	**NK2**	**NK3**

Chemotaxis	+++	-	-
Lymphocyte proliferation	+++	+	-
Increased neutrophil motility	+++	+	-
Monocyte/macrophage stimulation	++	+	-
Mast cell activation	++	+	-

Modified from Joos *et al. *[17]

## Conclusions

Overall we conclude that the number of studies on tachykinin receptors modulation in asthma is very small and limited both in sample size and patients characteristics, as well as in asthma outcome results. However, evidence on the role of tachykinin receptors antagonism in asthma has been gathered and their potential to regulate some airway functions has been recognized. Longer randomized trials standardized rating scales and outcome measures, at similar time periods and with similar antagonists are required. This further research will also be important to allow investigators to further define the role of SP and NKA in asthma pathogenesis.

## Competing interests

The authors declare that they have no competing interests.

## Authors' contributions

RR and AM designed the protocol. RR and NC performed the searches. RR performed the selection and data extraction. AM checked the data. RR drafted the manuscript. AM and RS revised the manuscript. All authors read and approved the final manuscript.

## Pre-publication history

The pre-publication history for this paper can be accessed here:

http://www.biomedcentral.com/1471-2466/11/41/prepub
